# LC‐DAD‐MS^n^ and HR‐LC‐QTOF Analysis of *Ziziphus budhensis* Leaves and Evaluation of Their In Vitro and In Vivo Biological Activities

**DOI:** 10.1002/cbdv.202402835

**Published:** 2025-06-16

**Authors:** Samjhana Bharati, Binita Maharjan, Timila Shrestha, Shyam Sharan Shrestha, Stefania Sut, Hari Prasad Devkota, Ram Lal Swagat Shrestha, Stefano Dall'Acqua

**Affiliations:** ^1^ Department of Chemistry Amrit Campus Tribhuvan University Kathmandu Nepal; ^2^ Himalayan Research and Development Center Kathmandu Nepal; ^3^ Department of Pharmaceutical and Pharmacological Sciences University of Padova Padova Italy; ^4^ Graduate School of Pharmaceutical Sciences Kumamoto University Kumamoto Japan

**Keywords:** bioactivities, in vivo, in vitro, LC‐DAD‐MS, *Ziziphus budhensis*

## Abstract

*Ziziphus budhensis* Bhattarai & M.L. Pathak is a specie described in 2015 in Nepal poorly studied for its phytochemical composition and possible bioactivities. In this study, six extracts were obtained from *Z. budhensis* leaves using ultrasound assisted extraction with solvents in increasing polarity namely hexane, dichloromethane, chloroform, ethyl acetate, acetone, and methanol. The phytochemical contents, along with the antioxidant, antibacterial, and antifungal capabilities, were evaluated for all the extracts to screen possible bioactivities. Chemical composition was studied combining liquid chromatography coupled with diode array and multiple stage mass spectrometry (LC‐DAD‐MS^n^) and liquid chromatography coupled with quadrupole time of flight high resolution mass spectrometry (HR‐MS‐QTOF). Forty‐six secondary metabolites belonging to the classes of phenolics, benzyl‐isoquinolinic alkaloids, cyclopeptide alkaloids, triterpene aglycone and saponins were identified. Among them 23 different derivatives were subjected to quantitative analysis and quercetin 3‐O‐β‐neohesperidoside (179.63 mg/g), quercetin‐3‐O‐rutinoside (123.20 mg/g), and quercetin‐3‐O‐rhamnoside (116.62 mg/g) were the most abundant in the methanol extract while quercetin 3‐O‐β‐neohesperidoside (236.06 mg/g), medicagenic acid (192.80 mg/g) were the most abundant in acetone extract and oleanolic acid (163.14 mg/g) was the most abundant in dichloromethane extract. To screen possible bioactivities extracts were tested against Gram‐positive (*Bacillus subtilis* and *Staphylococcus aureus*), Gram‐negative bacteria (*Escherichia coli* and *Klebsiella pneumoniae*), and *Candida albicans* and the ethyl acetate and acetone extracts presented significant activities. In vivo data were obtained using brine shrimp lethality test to evaluate the toxicity levels of the *Z. budhensis* leaves extracts. Acetone (LC_50_ = 145.04 µg/mL), methanol (50.22 µg/mL), and ethyl acetate (124.86 µg/mL) extracts exhibit toxic effects on nauplii, highlighting the importance of understanding extract‐specific toxicity profiles in ecological assessments. In addition, in vivo acute oral toxicity test on mice was performed on all six extracts and showed no toxic effects, suggesting their safety at tested doses during oral administration. The findings of this study advocate for further in‐depth research into the use of *Z. budhensis* leaves for medicinal purposes.

## Graphical abstract

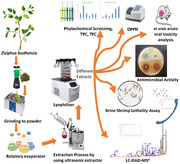

## 1 Introduction

Plants have been used by humans as medicine since antiquity and have been used and exploited as one of the most valuable sources of medicinal compounds. It is well‐known that the synthesis of secondary metabolites is related to genetic and environmental parameters, for this reason the investigation of endemic and unusual species can be of great interest in the discovery of bioactive compounds. Phytochemicals as phenolics, tannins, alkaloids, flavonoids, glycosides, and terpenoids have been largely used as lead compounds for the development of drugs and significant review considering the drugs discovered from natural sources have been published [1, 2, 3]. For these reasons the accurate study of plant species grown in particular pedoclimatic conditions can be of great interest for the discovery of new sources of bioactive constituents.

Nearly 200 species belonging to the genus *Ziziphus* (Rhamnaceae) have been reported [4]. In general, they are distributed around the world mostly in the subtropical and tropical parts of the Mediterranean region, Asia and America. These plants range from shrubs to small and medium‐sized trees, which may be upright or sprawling and frequently climbing. They can be evergreen or deciduous and are often spiny, with leaves arranged alternately [5]. *Ziziphus* is a genus that has been used for centuries in traditional medicinal all over the world to treat diseases like fever, ulcer, diarrhea, anxiety, anorexia, lassitude, diabetes, and skin infection [6, 7], furthermore several species produce edible fruits that are highly considered for their nutritional properties, and finally the utilization of *Ziziphus* leaf as a medicinal herbal tea has been extensively reported. Literature search reveal that the traditional medicines containing *Ziziphus* are claimed with multiple medicinal effects mostly as a sedative, hypnotic, anxiolytic, antitumor, and anti‐inflammatory [5, 8], but are also claimed for the effects in insomnia, diarrhea, pharyngitis, bronchitis, anemia, irritability, hysteria [4]. Some of the most documented activities are the anti‐ulcerogenic activity of *Ziziphus lotus*, the antiproliferative effects of *Ziziphus jujuba*, and the antimicrobial activity of *Ziziphus mauritiana* [4].

For example, *Z. jujuba*, a medium sized tree producing fruit similar to olive has been used both as a food and as medicinal species in several country of the world [4, 5]. For all the plant parts have been reported significant pharmacological activity. Roots of different plants of *Ziziphus* genus are used in the Ayurvedic to treat headaches, nausea, and coughing [9]. The fruit is claimed as an emollient, digestive, it ameliorates blood, has anti‐inflammatory and antipyretic properties. *Z. jujuba* was studied for its antioxidant, antibacterial, antihyperglycemic, hepatoprotective, and sedative properties as reported in a recent review [9]. The importance of *Z. jujuba* and related species in the traditional medicine of Nepal, India and other Asian countries as well as its importance in many socio‐economical aspect, for commerce and market have been extensively explained in a recent book chapter [10] showing the large impact of this specie on society and population.

The members of the *Ziziphus* genus are known for the production of large number of secondary metabolites including saponins, tannins, flavonoids, cyclopeptide alkaloids, and several classes of phenolic [5]. *Ziziphus* species have important role in traditional medicine and have been used to treat various diseases [5, 11, 12] including chronic inflammatory conditions for centuries [13].

While many species within this genus have been studied and confirmed for their traditional medicinal uses, numerous others still require investigation to establish their precise phytochemical composition, biological activities and their therapeutic potential [14]. In many cases the local population utilizes the fruits of this species as a food source. The leaves of *Ziziphus spina‐christi* possess high levels of total phenolic content (TPC) and significant total flavonoid content (TFC) [15]. Seven *Ziziphus* species have been identified in Nepal. *Ziziphus budhensis* Bhattarai & M. L. Pathak [16] (synonym *Ziziphus xiangchengensis* Y. L. Chen & P. K. Chou) was discovered in 2015. It is known by many names in Nepal as “Bodhichitta,” “Buddhamala,” or “Bodhi,” where “Bodichitta” means “mind of enlightenment” and also defined as a mind, motivated by compassion for all beings that spontaneously seek enlightenment. It is considered to be a part of Buddhism and is only known to exist in Nepal and China [16, 17].

*Z. budhensis* only grows naturally in the Timal area of the Kavrepalanchok District of Nepal at an elevation of 1000–2000 m. Its stem is glabrous, leaves are alternate, and flowers are yellow–green. It is commonly known as “Bodhichitta” which is revered for its religious significance, with its seeds used for meditation [18]. The tree holds significant religious and economic importance. Notably, the inner stony endocarps of the fruits are used by Buddhists to make rosary beads, a practice that has been in place for a long time [19]. Smaller seeds with more faces are used to make garland which is highly valued for its religious belief. The Buddhist people use wreaths made of beads for spiritual peace [16, 18, 20].

Nepal, due to its peculiar territory and pedoclimatic conditions, can be considered one of the very significant hot spots of biodiversity [21] and the investigation of endemic plant species can offer a unique opportunity for new discovery in the area of bioactive plant derived compounds.

**FIGURE 1:**
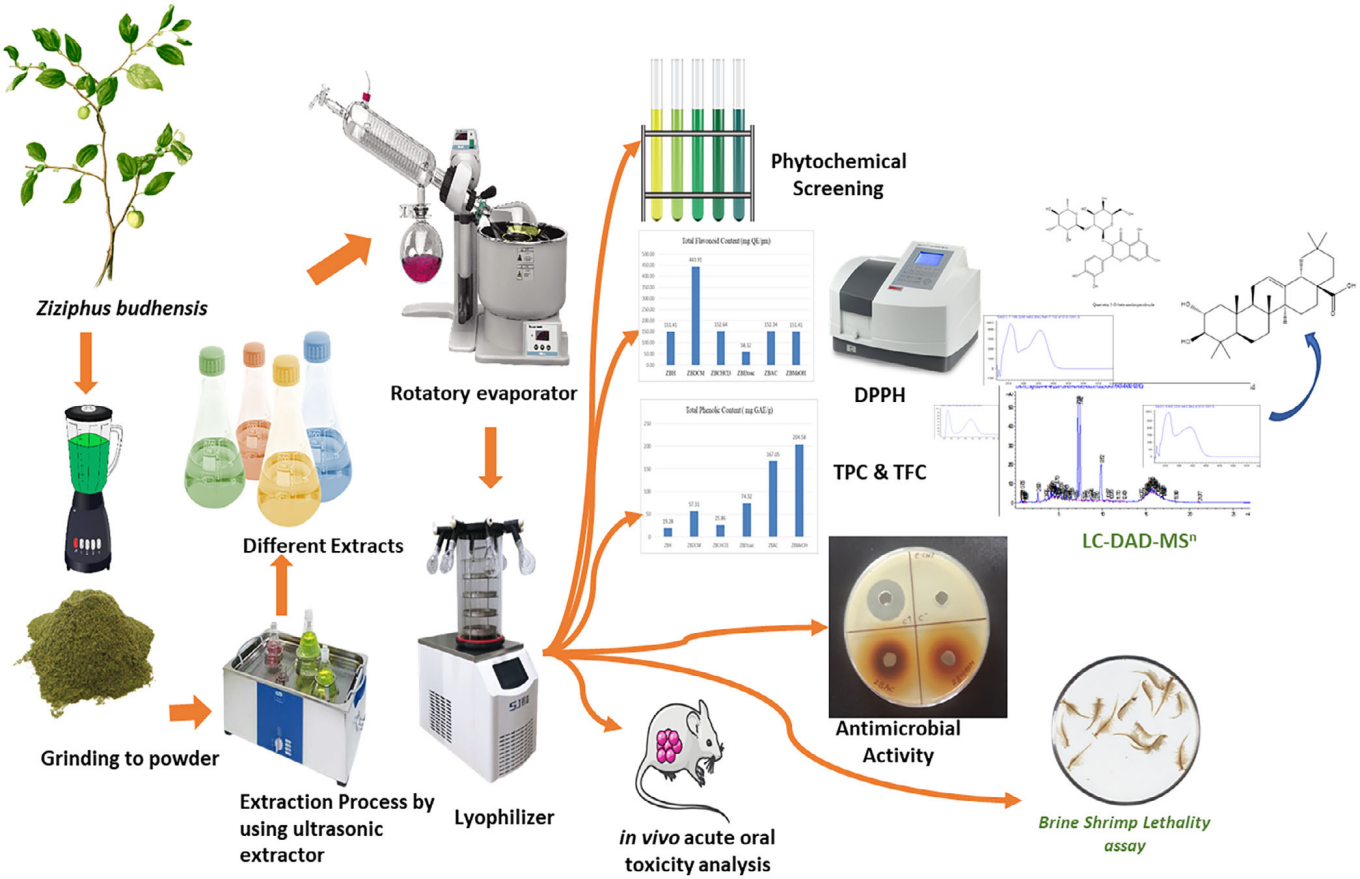
Overview of the research process.

This study was aimed to investigate the chemical composition and to evaluate biological activities, through the preliminary test like the TPC and TFC, along with the radical scavenging capacity and antimicrobial activities of leaf extracts from *Z. budhensis* collected from the Kavrepalanchok District of Nepal. To help future research the principal secondary metabolites present in the extract were also quantified and the safety of the extract through oral administration in mice was assessed (Figure 1).

## 2 Results and Discussion

### 2.1 Extracts and Phytochemical Screening

The *Z. budhensis* leaf extracts were obtained using ultrasound‐assisted solvent extraction (UAE) to have efficient extraction of the secondary metabolites but operating at room temperature. As a strategy to separate compounds with different polarity extraction was operated using different solvents. Extraction was started using hexane (ZBLH), dichloromethane (ZBLD), chloroform (ZBLC), ethyl acetate (ZBLE), acetone (ZBLA), methanol (ZBLM) in sequential approach. For each extraction step yields were calculated on the basis of the dry weight of plant materials and were 41.26% for ZBLH, 37.93% for ZBLD, 0.79% for ZBLC, 0.89% for ZBLE, 1.16% for ZBLA, and 3.18% for ZBLM, respectively. The selection of the different solvents can help to concentrate different classes of phytoconstituents in the different extracts due to the different solubility. To reveal the possible different constituent in the extracts obtained with the different solvents a phytochemical screening was performed with colorimetrical assays. The test showed positive results for phenols and flavonoids in all six extracts, whereas alkaloids were detected only in acetone and methanol extracts. Saponins were revealed in all five extracts, besides hexane, and tannins were present in methanol, chloroform, acetone, and ethyl acetate extracts. Terpenoids were present only in hexane and acetone extracts. The qualitative findings in the table are shown by the signs (+) and (−), which denote the existence and lack of phytochemicals, distinctly in Table 1. The results show that the use of a sequential extraction approach can be useful to extract different classes of compounds and revealed that acetone and methanol appear the most efficient solvents for all the different compounds while hexane and dichloromethane can be more selective on specific classes of phytoconstituents.

**TABLE 1:** Phytochemical screening of leaf extracts of *Ziziphus budhensis* (ZBL).

Phytochemicals	ZBLH	ZBLD	ZBLC	ZBLE	ZBLA	ZBLM
Alkaloids	−	−	−	−	+	+
Flavonoids	+	+	+	+	+	+
Phenol	+	+	+	+	+	+
Tannins	−	−	+	+	+	+
Terpenoids	+	−	−	−	+	−
Saponins	−	+	+	+	+	+

### 2.2 TPC and TFC Analysis

The TPC and TFC of extracts were determined using spectrophotometric techniques. The TPC was measured as milligrams of gallic acid equivalents. Likewise, the TFC per gram of dried material was expressed in milligrams of quercetin equivalents [22, 23].

**FIGURE 2:**
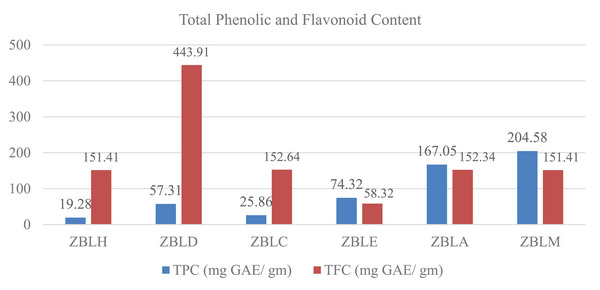
Total phenolic (TPC) and total flavonoid content (TFC) of the leaf extracts of *Ziziphus budhensis*.

The TFC was highest in the dichloromethane (ZBLD) extract, reaching 443.91 mg QE/g, while the lowest was recorded at 58.32 mg QE/g in the ethyl acetate extract (ZBLE). The TPC varied between 19.28 and 204.58 mg GAE/g, with the methanolic extract (ZBLM) showing the highest level of phenolics and the hexane extract (ZBLH) having the lowest among all extracts from *Z. budhensis*, as illustrated in Figure 2.

The TPC (204.58 mg GAE/g) and TFC (151.41 mg QE/g) in the methanolic extract differ significantly from the values (79.79 mg GAE/g and 62.85 mg QE/g) reported by Pandey et al. Variations in these measurements could be influenced by harvesting season, climate, and the sample's authenticity [24]. In a study by Hossain et al., the maximum TPC was detected in the ethyl acetate extract (190.80 ± 0.30 mg GAE/g), with the lowest in the hexane extract (48.46 ± 0.67 mg GAE/g), following the order of ethyl acetate > methanol > hexane [25]. In contrast, our study found the order to be methanol > acetone > ethyl acetate > dichloromethane > chloroform > hexane. Spectrophotometric assays can suffer from interference from other phytoconstituents nevertheless can be informative of the general extract's content and have been used as a starting screening approach to be further explored by more accurate and selective chromatographic and spectrometric approaches.

### 2.3 Antibacterial and Antifungal Screening Analysis

*Z. budhensis* extracts were evaluated for their antibacterial and antifungal effects at 100 mg/mL concentrations by Agar well diffusion method [26]. The tests were performed on two Gram‐positive bacteria (*Bacillus subtilis* ATCC 6051 and *Staphylococcus aureus* ATCC 6538P), two Gram‐negative bacteria (*Escherichia coli* ATCC 8739 and *Klebsiella pneumoniae* ATCC 700603), and the fungus *Candida albicans* ATCC 2091. The effectiveness of the extracts was evaluated by measuring the inhibition zones around the areas where the extracts were applied.

Table 2 shows that all the tested extracts present significant antimicrobial activities suggesting an important role in the search of new active compounds of *Z. budhensis* extracts. Getting more in the detail the ethyl acetate and acetone extracts had the highest antibacterial and antifungal activity among the tested extracts. Ethyl acetate extract was most effective against Gram‐positive *S. aureus* with a 1.6 cm inhibition zone. In contrast, acetone extract inhibited Gram‐negative *E. coli* and fungus *C. albicans* the most, with 1.8 cm zones. The tested doses were 400 µg/mL for the different extracts and 20 µg/mL for positive control (Khanamycin).

**TABLE 2:** Antibacterial activity of leaf extracts of *Ziziphus budhensis*.

Bacterial/fungal strain	Reference culture	Type	Positive control (c+) cm	Negative control (c−) cm	ZBLH ZOI (cm)	ZBLD ZOI (cm)	ZBLC ZOI (cm)	ZBLE ZOI (cm)	ZBLA ZOI (cm)	ZBLM ZOI (cm)
*Escherichia coli*	ATCC 8739	Gram‐negative	2.6	0	1.3	1.4	1.3	1.4	1.8	1.3
*Klebseilla pneumoniae*	ATCC 700603	Gram‐negative	1.8	0	1.2	1.3	0	1.4	1.4	1.3
*Bacillus subtilis*	ATCC 6051	Gram‐positive	2.6	0	1.1	1.2	1.1	1.5	1.5	1.3
*Staphylococcus aureus*	ATCC 6538P	Gram‐positive	2.6	0	1.2	1.3	1.1	1.6	1.4	1.3
*Candida albicans*	ATCC 2091	Fungi	2.4	0	1.3	1.4	1.3	1.8	1.8	1.5

Abbreviations: A, acetone; C, chloroform; D, dichloromethane; E, ethyl acetate; H, hexane; M, methanol.

Considering results, all the extracts present observable activity against the selected microorganisms. A comparison of the obtained zone of inhibition (ZOI) showed that the ethyl acetate and acetone extract of *Z. budhensis* present the higher antibacterial and antifungal activity compared to the other. The ethyl acetate extract demonstrated the largest ZOI against *S. aureus*. Acetone extract showed a maximum ZOI against *E. coli* (1.8 cm). Both the extracts showed maximum ZOI against *C. albicans* fungi (1.8 cm). This preliminary screening is a starting point to study these plant extract's potential antibacterial and antifungal activity. There is a need for new antibacterial and antifungal compounds and the exploration of plant biodiversity can offer new opportunities. The observed data show the potential usefulness of *Z. budhensis* leaves extract in this field and point out the need for an accurate chemical analysis of the extracts to identify the phytochemicals involved in the observed activities.

### 2.4 Brine Shrimp Lethality Test

**FIGURE 3:**
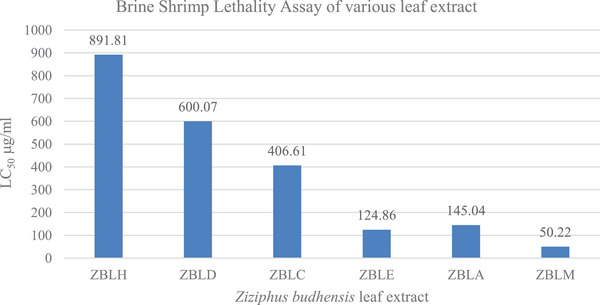
Brine shrimp lethality assay of the leaf extracts of *Ziziphus budhensis*.

The LC_50_ values for hexane, dichloromethane, chloroform, ethyl acetate, acetone, and methanol, extracts of *Z. budhensis* leaves were determined by exposing 20 freshly hatched nauplii to varying concentrations (10, 50, 100, 500, 800, and 1000 µg/mL) of each extract. The LC_50_ values were found to be 891.81, 600.07, 406.61, 124.86, 145.04, and 50.22 µg/mL, respectively. These findings illustrate a direct correlation between the concentration of the extract and the resulting mortality percentage (Figure 3). Mortality in nauplii was observed starting at a concentration of 500 µg/mL, with a mortality rate of 61% recorded at the highest concentration of 1000 µg/mL for the plant's hexane extract. The LC_50_ value of the hexane extract from *Z. budhensis* leaves was determined to be 891.81 µg/mL, indicating its nontoxic nature.

In the case of the dichloromethane extract, nauplii mortality started at a concentration of 500 µg/mL, and the mortality rate increased to 93% at the maximum concentration of 1000 µg/mL. The LC_50_ value of the dichloromethane extract from *Z. budhensis* leaves was determined to be 600.07 µg/mL, indicating its nontoxic nature. Similarly, nauplii began to perish at a concentration of 100 µg/mL for the chloroform extract, with a maximum mortality of 100% observed at 1000 µg/mL of the extract. The LC_50_ was found to be 406.61 µg/mL.

For the acetone extract, nauplii mortality began at a concentration of 10 µg/mL, and the LC_50_ of the acetone extract was determined to be 145.04 µg/mL. Concerning the methanol and ethyl acetate extracts of the plant LC_50_ of methanol and ethyl acetate were found 50.22 and 124.86 µg/mL, indicating the cytotoxic nature of these extracts. At a concentration of 100 µg/mL, complete mortality was seen in both instances.

### 2.5 Antioxidant Screening Analysis

**FIGURE 4:**
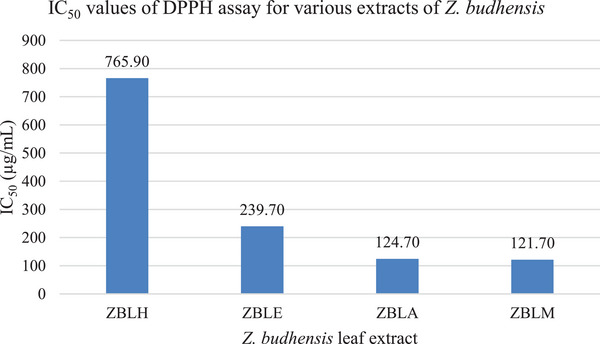
Antioxidant assay of leaf extracts of *Ziziphus budhensis*.

The methanolic extract of *Z. budhensis* exhibited the strongest free radical scavenging activity, with an IC_50_ value of 121.70 µg/mL. Our results are completely consistent with a study by Pandey et al., which reported the antioxidant activity as represented by an IC_50_ value of 134.69 ± 1.97 µg/mL [24]. The strong antioxidant capabilities of the methanolic, acetone, and ethyl acetate extracts from *Z. budhensis* correlated closely with their respective TPC levels, as shown in Figure 4. Various studies have confirmed the link between phenolic content and antioxidant activity. For example, Sengul et al. emphasized the importance of phenolic compounds in plant defense, particularly in reducing the formation of reactive oxygen species (ROS) [27]. The presence and levels of phenolic and flavonoid compounds in the extracts likely account for the typical antioxidant effects observed in these extracts. Essentially, this implies that the concentration of these substances in the extracts directly affects their capacity to counteract free radicals, consistently offering antioxidant advantages.

### 2.6 In Vivo Acute Oral Toxicity Study

The in vivo oral acute toxicity test of all six extracts on mice showed no toxicity during the oral administration at a dose of 2000 mg/kg. The data obtained revealed that all extracts could be considered safe for medicinal purposes (Table 3 and Table 4).

**TABLE 3:** Median lethal dose (LD_50_) of different leaf extracts of *Ziziphus budhensis*.

Extracts	LD_50_ (mg/kg BW)	Hazard statement	Remarks
ZBLH	> 2000 mg/kg BW	It could be dangerous if ingested	No fatalities were observed at a dose of 2000 mg/kg
ZBLD	> 2000 mg/kg BW	It could be dangerous if ingested	No fatalities were observed at a dose of 2000 mg/kg
ZBLC	> 2000 mg/kg BW	It could be dangerous if ingested	No fatalities were observed at a dose of 2000 mg/kg
ZBLE	> 2000 mg/kg BW	It could be dangerous if ingested	No fatalities were observed at a dose of 2000 mg/kg
ZBLA	> 2000 mg/kg BW	It could be dangerous if ingested	No fatalities were observed at a dose of 2000 mg/kg
ZBLM	> 2000 mg/kg BW	It could be dangerous if ingested	No fatalities were observed at a dose of 2000 mg/kg

### 2.7 Phytochemical Composition Studied by Liquid Chromatography Diode Array Multiple Stage Mass Spectrometry and High‐Resolution Quadrupole Time of Flight Mass Spectrometry

The preliminary assays on the phytochemical composition indicated the possible presence of different classes of phytoconstituents (Table 1) and revealed the significant antibacterial and antifungal activities of the extracts. For these reasons detailed investigations were performed using a combination of multiple‐stage mass spectrometry (MS^n^) by ion trap mass analyzer and high‐resolution mass spectrometry by quadrupole time of flight (QTOF) both coupled with liquid chromatography. Furthermore, diode array detector was used in parallel to mass spectrometers to reveal the presence of phenolics and alkaloids that can present significant UV absorption. The liquid chromatography diode array multiple stage mass spectrometry (LC‐DAD‐MS^n^) analysis allowed to detect many classes of secondary metabolites in the *Z. budhensis* extracts. The diode array chromatograms showed peaks presenting UV spectra resuming the flavonol structure with a maximum absorption at 350–370 nm and some other characterized by a single maximum at 280 nm supporting the structure of flavan‐3‐ols. Other peaks present UV spectrum with a maximum at 290 nm and can be ascribed to protocatechuic acid. MS^n^ in negative ion mode allowed the identification of several compounds that belong to the classes of protocatechuic acid derivative, flavonol‐3‐ols, procyanidin B‐type dimer, and (epi)afzelech‐(epi)catechin. Furthermore, flavonols were identified, mainly quercetin and kaempferol derivatives, with different glycosylation patterns. Some of the flavonols presented *O*‐glycosylation as revealed in negative ion mode by the formation in MS^2^ of the ion corresponding to the aglycone and neutral losses of 162 Da, for hexoside, 308 Da for rutinoside, 146 Da for deoxyhexoside moiety, respectively. The study of the fragmentation pattern by ion trap allows the identification metabolites with same *m*/*z* as parent ion but diverse structure, namely quercetin‐3‐O‐rutinoside and quercetin 3‐O‐β‐neohesperidoside. Furthermore, two C‐glycoside derivatives of apigenin, namely spinosyn and isospinosin, were detected thanks to the typical fragmentation of the C‐glycoside moiety and confirmed by standard comparison. Assignments were confirmed by the measurement in QTOF of HR‐MS and by the observation of diagnostic fragments by MS^e^ as well as by the comparison with reference standards.

A series of peaks were observed in positive ion mode and they presented in the HR‐MS the identical molecular formula reported for several cyclopeptide alkaloids that were recently identified by LC‐MS approach from *Ziziphus* leaves. In that paper authors also reported significant information's about their fragmentation in ESI [28] allowing easy identification by the comparison of the data. These compounds are macrocyclic polyamides possessing a 13‐, 14‐ or 15‐atom ring formed by a styrylamine moiety, a β‐hydroxy amino acid and other amino acid moieties forming a side chain. They can be classified on the basis of ring dimension and the number of building subunits (4 or 5). In our samples the observed alkaloids are both presenting 14 atom ring and are belonging to the 5(14)‐amphibine‐B type namely the Mauritine F and the 5(14)‐scutianine‐A class namely the oxyphylline A and its derivative [28].

These alkaloids are considered as one of the main chemotaxonomic markers in Rhamnaceae family and are important for the *Ziziphus* genus [28, 29].

Other compounds have been observed in positive ion mode and the benzylisoquinoline alkaloids were identified. The peak with molecular ion [M+H]^+^ at *m*/*z* 288.3131 corresponding to the molecular formula of C_17_H_15_NO_3_ showing a fragment at *m*/*z* 188 and 156 supporting the presence of juzirine [30]. A second peak showing molecular ion [M+H]^+^ at *m*/*z* 298.10797 was ascribed tentatively to another benzoquinoline alkaloid derivative namely the annocherine A. Finally, two aporphine alkaloids in particular boldine and isoboldine were also identified [31, 32].

A series of triterpene acid as, for example, maslinic and oleanolic were identified as aglycone, but also some derivatives were identified as glycosidic saponins [8]. Triterpene of ceanothane‐type were identified and this latter have been exclusively isolated from *Ziziphus* genus and some other Rhamnaceae plants [33]. Reference compounds when available were also injected to corroborate identification and summary of identified compound is reported in Table 5.

Extraction with the different solvents using sequential approach allowed a partial fractionation of constituents showing that the flavonoid derivatives and the flavan‐3‐ols as well as the cyclopeptide alkaloids were more efficiently extracted in the more polar solvents while the triterpene acid were also detected in the hexane, dichloromethane, and chloroform extracts. All qualitative results are summarized in Table 5.

**TABLE 4:** Classification of substances following the guidelines of the third edition of the Globally Harmonized System for Classification and Labeling of Chemicals (GHS) [61].

Ranges (mg/kg)	Category	Classification	Hazard statement
> 2000 mg/kg	Category 5	Unclassified	Could be dangerous if ingested
> 300–≤ 2000 mg/kg	Category 4	Hazardous	Harmful if ingested
> 50–≤ 300 mg/kg	Category 3	Toxic	Toxic if ingested
> 5–≤ 50 mg/kg	Category 2	Extremely toxic	Deadly if ingested
< 5 mg/kg	Category 1	Severely toxic	Deadly if ingested

**TABLE 5:** Compounds observed in LC‐DAD‐MS and high‐resolution quadrupole time of flight (QTOF) mass spectrometry of leaf extracts of *Ziziphus budhensis*, letters in extract column indicate the different extracts obtained with the various solvents (H, hexane; D, dichloromethane; C, chloroform; E, ethyl acetate; A, acetone; M, methanol).

S.N	rt	M+H HR	QTOF fragments	M−H	IT fragments	Identified compounds	Formula	Class of compound	Extracts
1	5.18	579.15039	465.10123	577	425 407 389 289	Procyanidin dimer B type	C_30_H_26_O_12_	Flavan‐3‐ol	E, A, M
2	5.57	291.0868		289	245 227 203 175	Catechin*	C_15_H_14_O_6_	Flavan‐3‐ol	E, A, M
3	5.8	328.15537				Isoboldine	C_19_H_21_NO_4_	Aporphyne alkaloid	A, M
4	5.84	282.3131	188.0713 156.004			Juzirine	C_17_H_15_NO_3_	Benzylisoquinoline alkaloid	A, M
5	5.85	328.1543				Boldine*	C_19_H_21_NO_4_	Aporphyne alkaloid	E, A, M
6	6.08	298.10797	176.0708			Annocherine A	C_17_H_15_NO_4_	Benzylisoquinoline alkaloid	E,A,M
7	6.21	291.0868		289	245 227 203 175	Epicatechin*	C_15_H_14_O_6_	Flavan‐3‐ol	A, M
8	6.7	611.16229	303.0506	609	301 271 257 255 179	Quercetin‐3‐O‐rutinoside*	C_27_H_30_O_16_	Flavonol	A, M
9	6.76	611.15612	303.05106	609	447 301 271 179	Quercetin 3‐O‐β‐neohesperidoside*	C_27_H_30_O_16_	Flavonol	A, M
10	6.8	465.10248	303.0505	463	301 271 255	Quercetin‐3‐O‐glucoside*	C_21_H_19_O_12_	Flavonol	A, M
11	7.06	595.1663	287.0551	593	447 285 255	Kaempferol‐3‐O‐β‐neohesperidoside	C_27_H_30_O_15_	Flavonol	A, M
12	7.12	595.1663	287.0551	593	285 255	Kaempferol‐3‐O‐rutinoside*	C_27_H_30_O_15_	Flavonol	A, M
13	7.14	451.1197	287.0566	449	287 269 259 215	Eryiodichtiol‐7‐O‐glucoside*	C_21_H_22_O_11_	Flavanone	A, M
14	7.15	449.10784	303.0505	447	301	Quercetin‐3‐O‐rhamnoside*	C_21_H_20_O_11_	Flavonol	A, M
15	7.16	595.1663	303.0505	593	447 301 271 179	Quercetin‐dirhamnoside	C_27_H_30_O_15_	flavonol	A, M
16	7.19	317.06473	153.01862	315	153	Protocatechuic acid glucoside	C_13_H_16_O_9_	Phenolic acid	A, M
17	7.58	897.5206	735.5401			Jujubasaponin I	C_47_H_76_O_16_	triterpene saponin	E, A, M
18	8.041	913.51533	895.455			Christinin A/C	C_47_H_76_O_17_	triterpene (dammarane)saponin	E, A, M
19	8.06	757.2192	303.0506	755	609 301 271 179	Quercetin‐3‐O‐rutinoside‐deoxyhexoside	C_33_H_40_O_20_	Flavonol	A, M
20	8.1	943.52609	781			Jujubasaponin IV/V	C_48_H_78_O_18_	triterpene saponin	E, A, M
21	8.1	619.39931	439.3567			3‐O‐Z‐p‐Coumaroylalphitolic acid	C_39_H_54_O_6_	triterpene‐hydroxycinnamic ester	E, A, M
22	8,11	581.15066	303.0506	579	301 271	Quercetin‐pentosyl‐deoxyhexoside	C_26_H_28_O_15_	Flavonol	A, M
23	8.23	787.1934	303.0506	785	755 609 301 271 179	Quercetin‐3‐O‐rutinoside‐glucuronide	C_33_H_38_O_22_	Flavonol	A, M
24	8.3			431	385 (formic) 153	Protocatecuic acid hexoside derivative		Phenolic acid	A, M
25	8.67	563.15514	289.1068	561	435 407 289	(epi)afzelech‐(epi)catechin	C_30_H_26_O_11_	Flavan‐3‐ol	A, M
26	8.72	939.5293	455 439			Jujubasaponin II/III	C_49_H_78_O_17_	Triterpene saponin	A, M
27	8.79	619.39932	439.3566			3‐O‐Z‐p‐Coumaroylmaslinic acid	C_39_H_54_O_6_	Triterpene‐hydroxycinnamic ester	A, M
28	9.84	939.5294	455			Jujubasaponin II/III isomer	C_49_H_78_O_17_	Triterpene saponin	A, M
29	10.07	609.1814	449.1079 303,0526	607	301	Spinosin	C_28_H_32_O_15_	Flavonol	E, A, M
30	10.3			517	455 407	Oleanolic/ursolic acid derivative		Triterpene	E, A, M
31	10.47	716.3442	698 610			Oxyphylline A	C_42_H_45_N_5_O_6_	Cyclopeptide alkaloid	A, M
32	10.53	716.3432				Oxyphylline A isomer	C_42_H_45_N_5_O_6_	Cyclopeptide alkaloid	A, M
33	10.7	562.3024	378.18079			Mauritine F	C_31_H_39_N_5_O_5_	Cyclopeptide alkaloid	A, M
34	10.81	609.1814		607	301	Isospinosin	C_28_H_32_O_15_	flavonol	E, A, M
35	10.89	473.3631		471	409 377	Maslinic acid*	C_30_H_48_O_4_	Triterpene acid	D, C, E, A, M
36	10.89	981.54174				Ziziphyn	C_51_H_80_O_18_	Triterpene saponin	A, M
37	11.41	317.06473	153.01862	315	153	Protocatechuic acid glucoside	C_13_H_16_O_9_	Phenolic acid glicoside	E, A, M
38	11.45	449.10797	303.0506	447	301 271 179	Quercetin‐3‐O‐rhamnoside*	C_21_H_20_O_11_	Flavonol Glicoside	A, M
39	11.91	503.3371		501	457 439 413	Medicagenic acid	C_30_H_46_O_6_	Triterpene acid	H, D, C, E, A, M
40	12.4	471.34989	453.3365			Ziziberanalyc acid	C_30_H_46_O_4_	Triterpene acid	H, D, C, E, A, M
41	12.8	489.358001		487	423 371	Euscaphyc acid	C_30_H_48_O_5_	Triterpene acid	H, D, C, E, A, M
42	12.81	487.3325				Daechuine S5/Hovenine A/Discarine F	C_27_H_42_N_4_O_4_	Cyclopeptide alkaloid	H, D, C, E, A, M
43	13.18	457.3676				Oleanolic acid*	C_30_H_48_O_3_	Triterpene acid	H, D, C, E, A, M
44	13.6	471.34989	453.3365			Pomolic acid	C_30_H_46_O_4_	Triterpene acid	D, C, E, A, M
45	14.1	487.3418	423.32602			Ceanothic acid	C_30_H_48_O_3_	Triterpene acid	H, D, C, E, A, M
46	15.96	455.31559				ceanotheic acid	C_29_H_42_O_4_	Triterpenoid acid	H, D, C, E, A, M

The combination of the ion trap and the QTOF and the use of positive and negative ion mode allowed the identification of 46 different compounds. The data obtained from the preliminary screening were corroborated by LC‐MS analysis allowing the identification of flavonol, phenolics, alkaloids, saponins, cyclopeptide alkaloids and triterpenoids aglycone. Quantitative analysis on some of the identified compounds were performed and the results are reported as mg/g of dried extract and are summarized in Table 5 and 6.

**TABLE 6:** Quantitative determination of selected phytoconstituents in the leaf extracts of *Ziziphus budhensis*, letters in extract column indicate the different extracts obtained with the various solvents, compounds indicated with * have been confirmed by standard injection.

Identification	ZBLH (mg/g)	ZBLD (mg/g)	ZBLC (mg/g)	ZBLE (mg/g)	ZBLA (mg/g)	ZBLM (mg/g)
Protocatecuic acid glucoside	—	—	—	0.18 ± 0.02	2.93 ± 0.10	3.92 ± 0.25
Procyanidin dimer B	—	—	—	2.05 ± 0.02	22.2 ± 1.02	1.4 ± 0.05
Catechin*	—	—	—	3.21 ± 0.08	10.2 ± 0.80	3.07 ± 0.10
Epicatechin*	—	—	—	0.30 ± 0.02	0.32 ± 0.02	0.35 ± 0.03
Quercetin‐3‐O‐rutinoside*	—	—	—	14.32 ± 0.95	2.60 ± 0.02	123.2 ± 2.25
Quercetin 3‐O‐β‐neohesperidoside	—	—	—	11.39 ± 0.99	236.06 ± 2.32	179.63 ± 2.12
Quercetin‐3‐O‐glucoside*	—	—	—	1.05 ± 0.10	18.34 ± 1.00	3.06 ± 0.22
Kaempferol‐3‐O‐βneohesperidoside	—	—	—	1.71 ± 0.09	15.14 ± 0.99	9.91 ± 0.71
Kaempferol‐3‐O‐rutinoside*	—	—	—	1.03 ± 0.03	21.76 ± 0.95	6.10 ± 0.12
Eryidichtiol‐7‐O‐glucoside*	—	—	—	—	8.98 ± 0.22	1.93 ± 0.06
Quercetin‐3‐O‐rhamnoside*	—	—	—	14.06 ± 0.82	2.59 ± 0.08	116.62 ± 0.98
Quercetin‐dirhamnoside	—	—	—	4.32 ± 0.11	27.26 ± 0.62	12.42 ± 0.44
Protocatecuic acid hexoside derivative	—	—	—	4.35 ± 0.06	11.98 ± 0.65	10.65 ± 0.55
Quercetin‐3‐O‐rutinoside‐deoxyhexoside	—	—	—	3.24 ± 0.04	41.64 ± 0.88	5.53 ± 0.52
Quercetin‐pentosyl‐deoxyhexoside	—	—	—	—	1.31 ± 0.04	3.54 ± 0.06
Quercetin‐3‐O‐rutinoside‐glucuronide	—	—	—	1.52 ± 0.05	25.59 ± 0.09	2.56 ± 0.07
(Epi)afzelech‐(epi)catechin	—	—	—	—	7.56 ± 0.09	0.65 ± 0.02
Spinosin	—	—	—	6.25 ± 0.10	13.21 ± 0.23	14.32 ± 0.22
Isospinosin	—	—	—	0.35 ± 0.02	0.65 ± 0.03	1.32 ± 0.05
Maslinic acid*	18.08 ± 0.66	12.46 ± 0.44	16.20 ± 0.59	10.82 ± 0.25	38.64 ± 0.44	32.16 ± 0.32
Medicagenic acid	15.68 ± 0.35	100.79 ± 1.09	66.23 ± 0.89	129.31 ± 1.01	192.8 ± 1.31	130.39 ± 1.42
Euscaphyc acid	—	1.79 ± 0.085	1.85 ± 0.11	2.56 ± 0.32	17.35 ± 0.15	3.28 ± 0.06
Oleanolic acid*	—	163.14 ± 1.22	9.37 ± 0.55	5.21 ± 0.44	38.41 ± 0.19	15.32 ± 0.19
Total triterpene	33.8	278.2	93.7	148.0	287.2	181.2
Total phenolic				69.3	470.3	500.2

As we can observe from the table the more lipophilic solvents, hexane, chloroform, and dichloromethane, are efficient in the extraction of the triterpene acid. The dichloromethane extract presents high content of triterpene mostly oleanolic and medicagenic acids compared to the other showing the possible application of this approach to obtain extracts with high content these bioactive compounds. We can see from the quantitative table that the same compounds are also extracted in high amount also using ethyl acetate, acetone and methanol but the more polar solvents compared to the dichloromethane are able to extract many other classes of secondary metabolites. So the lipophilic solvents hexane and chloroform can be used for a selective extraction of the triterpene because these solvents are not able to extract the cyclopeptide alkaloids and the phenol glycosides.

The polyphenols are partly extracted by ethyl acetate and are then extracted in a more efficient way by acetone or methanol with higher amount in this latter (Table 5). The extraction of some of the compounds is strongly influenced by the selected solvent. For example, procyanidin derivatives and catechin were efficiently extracted with acetone compared with ethyl acetate or methanol. This is probably due to the better solubility of flavan‐3‐ols on acetone compared to other solvents. On the other hand, all the flavonols and glycosidic derivatives of the phenolics are efficiently extracted using methanol rather than the other solvents.

The phytochemical composition of *Z. budhensis* leaves, reported in this work revealed a complex pattern of constituents. *Ziziphus* species are largely studied for their phytochemical constituents and our data show the presence of flavan‐3‐ols, flavonols, benzylisoquinoline alkaloids, cyclopeptide alkaloids, triterpene as, for example, recently shown by Sakna et al. that used LC‐MS to compare different species [8]. Nepalese *Z. budhensis*, leaves contain large amount of quercetin 3‐O‐β‐neohesperidoside, quercetin‐3‐O‐rutinoside, and quercetin‐3‐O‐rhamnoside and medicagenic acid as well as oleanolic acid.

## 3 Discussion

Considering the bioassays data we observed that all the obtained extracts revealed significant antibacterial and antifungal activities, suggesting that different classes of constituents may act on the microorganisms with different mechanisms. The possible role of the combination of different phytochemicals in the antibacterial activity can be of great interest. Plant extracts with complex chemical composition can show antimicrobial effects suggesting that the combination of various classes of compounds may play an advantage compared to the single constituent. Our results show that ethyl acetate and acetone extracts present the higher antimicrobial effects compared to the other tested extracts, and these two extracts present high levels of some triterpene acids as well as of flavan‐3‐ol derivatives as catechin and procyanidin, so we can argue that the combination of these two classes of compounds may be favorable for the observed antimicrobial effect. Recently, a paper demonstrated the synergistic antimicrobial activity of the triterpenoid acids, namely ursolic and oleanolic acid, and the flavonoid dihydromyricetin, suggesting that the combination of flavonoids and triterpenoid enhanced the antimicrobial effect [34]. The ethyl acetate presented in our preliminary screening the higher activities towards the studied microorganisms suggesting that a combination of the triterpene and the polyphenols can play a role in the antimicrobial effects. Acetone and methanol extract also present significant antimicrobial effects, their composition present higher content of polyphenols compare to the triterpene.

As a general consideration on the antimicrobial effects of *Z. budhensis* leaves extracts, the presence of terpenes, phenolics, flavonoids, alkaloids is valuable. These classes of compounds present membrane disruption as their most common mechanism of antimicrobial activity as recently reviewed [35]. However, the utilization of *Z. budhensis* leaves as antimicrobial agent obviously need to be investigated deeply, for example, using new omics approaches and network pharmacology to find the most effective extraction approach and combination of phytochemical that can be obtained from plant material as well as the possibility of using the extracts alone or in combination with actual antibiotics.

The high amounts of quercetin, procyanidins and catechin in the more polar extracts explain the higher antioxidant activity observed for the extracts. The tested dose in the performed antimicrobial assays was 400 µg/mL and the observed significant effect is particularly of interest for the more lipophilic extracts. To collect further data useful for the possible future evaluation of *Z. budhensis* extracts the exploration of preliminary toxicological aspect were evaluated by the brine shrimp lethality test. The extracts revealed limited toxicity for the hexane, dichloromethane and chloroform extracts all presenting LD_50_ > 400 µg/mL, and this value is comparable to the dose that revealed significant antimicrobial activities in the in vitro assay. The other extracts namely the ethyl acetate, acetone and methanol all present LD_50_ ranging from 145 to 50 µg/mL in the brine shrimp lethality test suggesting a moderate cytotoxicity of the extracts. To deeply investigate these preliminary toxicological aspects the acute toxicity was assessed in vivo on mice and response revealed limited toxicity against the tested animal model with no toxicity behavior during the oral administration up to dose of 2000 mg/kg. This encourage the further study and development of *Z. budhensis* as ingredient of health promoting products or herbal medicine.

**FIGURE 5:**
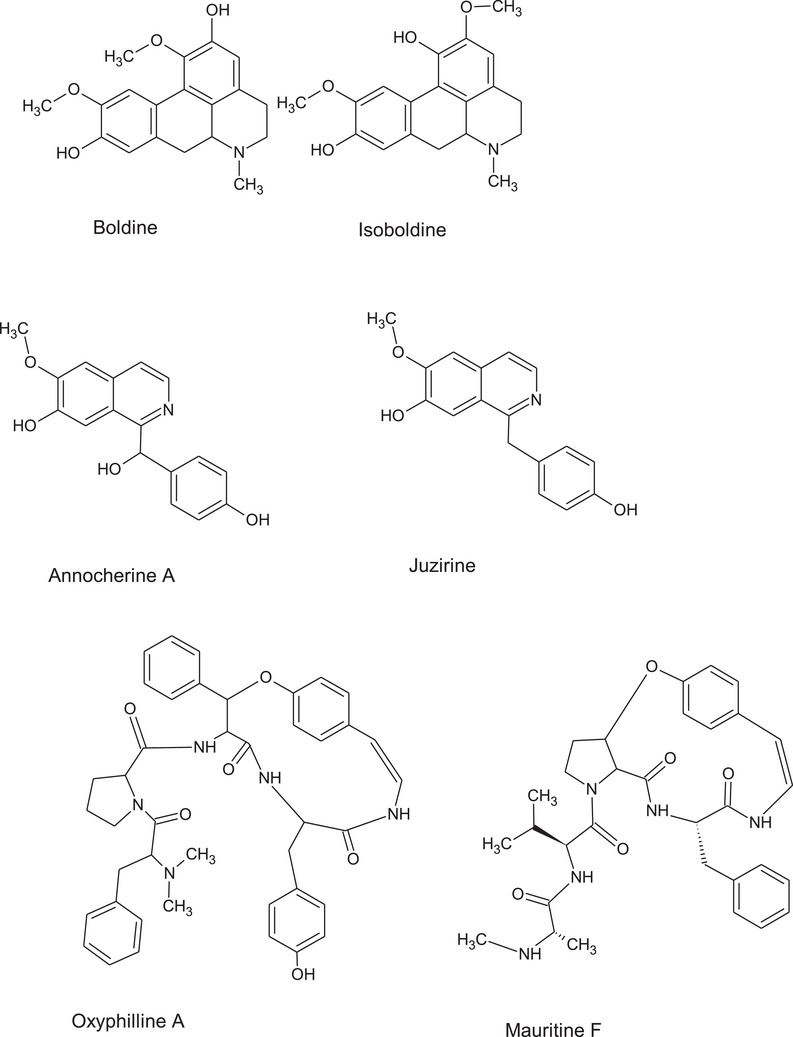
Structures of the most significant identified alkaloids in ziziphus budhiensis.

To complete the view of the possible usefulness of this plant species, accurate chemical characterization is needed. The phytochemical composition of *Z. budhensis* leaves extracts is here described for the first time. Considering the different classes of identified constituents, we can underline the presence of different classes of alkaloids. At first the aporphine alkaloids boldine and isoboldine (Figure 5) can be highlighted due to the relevant bioactivities reported for these compounds. Boldine bioactivities were recently reviewed showing its possible application as medicinal for nervous system injuries and neurodegenerative disorders, liver disease, inflammation, infections, and cancer [31]. Thus, *Z. budhensis* leaves extracts can be significant sources of this pharmacologically active compound. For the benzilisoquinoline alkaloids juzirine and annocherine A (Figure 5) no significant reports have been so far published on their potential bioactivities. Nevertheless, alkaloids of this class are now under investigation as potential hypocholesterolemic agents and shown significant activity in some in vitro models on the low‐density lipoprotein receptor (LDL‐R) and on the proprotein convertase subtilisin/kexin type 9 (PCSK9) pathway [36, 37]. The identification of some cyclopeptide alkaloids suggests the possibility to isolate or extract some of these compounds. Up to now the information about their possible bioactivities are limited but in previous paper some *Ziziphus* cyclopeptides have shown antiplasmodial effects [38].The isolation of cyclopeptide alkaloids from *Z. apetala* lead to the identification of the derivative mauritine A as a selective inhibitor of 11β‐hydroxysteroid dehydrogenase type the enzyme that reduces cortisone to cortisol [39].

**FIGURE 6:**
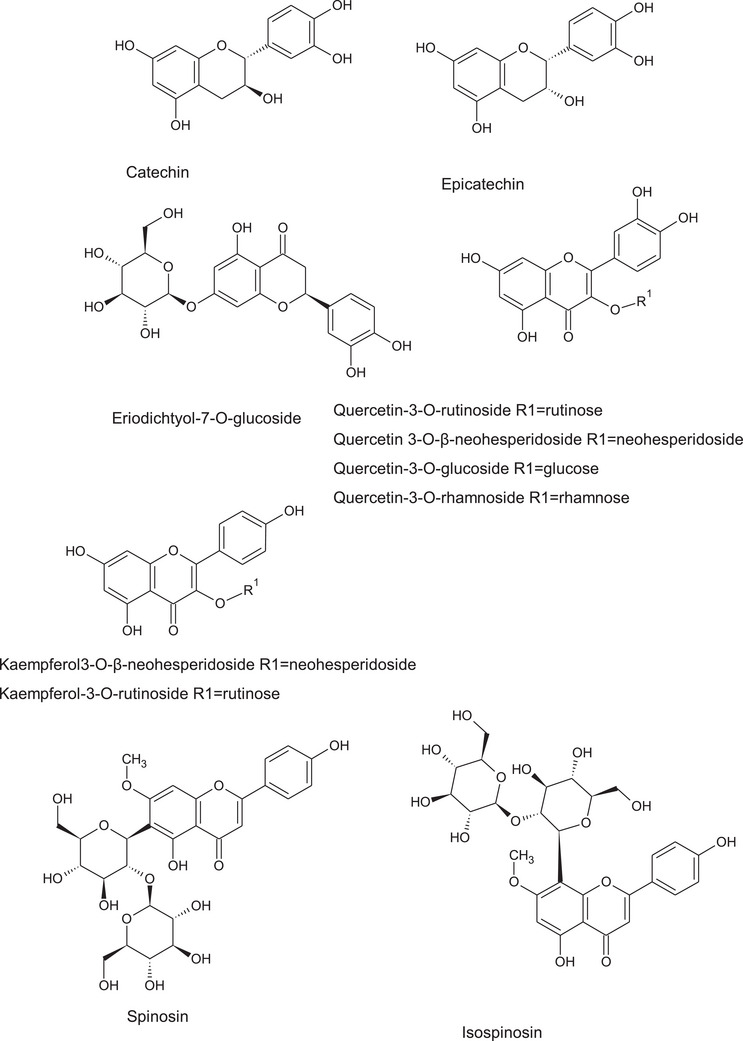
Structure of the most significant Identified phenolics in ziziphus budhiensis.

The significant presence of different classes of phenolics (Figure 6) makes the *Z. budhensis* leaves extracts very attractive for the nutraceutical and food supplement application thanks to the presence of catechin, epicatechin and many O‐ and C‐glycosidic flavonoids. Quercetin derivatives are very important natural products and have been extensively studied among the flavonols family due to the potent pharmacological effects. Glycosidic derivatives of quercetin have shown significant biological activities, as antioxidant, anti‐inflammatory, antibacterial, antifungal, antiviral, antidiabetic, anti‐obesity, anti‐Alzheimer, ion‐chelating, and many others. Glycosylation modulates quercetin solubility and in general quercetin glycosides have an improved water solubility than their aglycon form, leading to the result that some glycosides can present improved bioactivity than free quercetin. A complete review on quercetin glycoside bioactivities have been recently published and show the importance of these compounds in the possible development of medicinal or health promoting products [40]. In this regard, the the *Z. budhensis* leaves extracts can be considered as a good source of these bioactive flavonoids.

**FIGURE 7:**
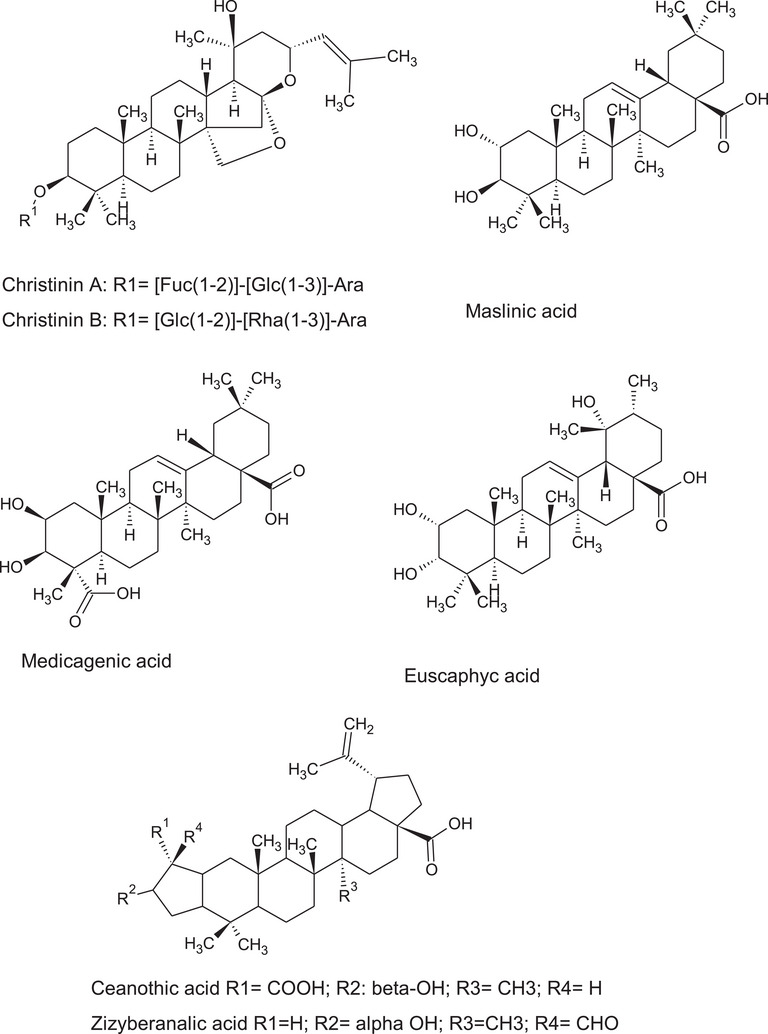
Structure of the most significant triterpenoids identified in ziziphus budhiensis.

Furthermore, the large number of identified triterpene acids (Figure 7) as well as triterpene saponins make this extract very attractive thanks to the high anti‐inflammatory activities [41] and immunomodulatory properties [42] of the triterpene acid derivatives that make this leave extract of great interest for the development of new drugs or new nutraceuticals targeted on inflammation and immunity. Considering the different triterpene acid identified in the *Z. budhensis* leaves extracts the presence of oleanolic acid can be highly evaluated. The oleanolic acid have been indicated as anti‐inflammatory agent able to activate the pituitary‐adrenal cortical system, inhibiting the synthesis or release of prostaglandins (PGs), inhibiting endotoxin‐mediated release of HMGB1 by endothelial cells or regulating MAPK, PI3K/Akt/NF‐κB/ICAM‐1/JAK/STAT signaling pathways [43]. Also, pomolic acid have been identified and this compound was extensively studied especially for its anticancer activities especially targeted on breast cancer and leukemia. A wide array of other pharmacological properties of pomolic acid have been reported indicating this constituent as important bioactive compound in *Z. budhensis* [44]. Thus, the extraction of *Z. budhensis* leaves with a polar solvent may result in high concentrated triterpene material that can be useful for the purification of such compounds.

The obtained data offer starting point for the further exploitation of *Z. budhensis* as source of natural bioactive compounds. Chemical results revealed the presence of multiple classes of phytoconstituents and biological assays shown significant although preliminary bioactivities.

The obtained phytochemical fingerprint can be used to guide further studies focused on the isolation of the different secondary metabolites to perform bioassays on purified chemicals. Furthermore, the knowledge about the different classes of compounds can guide specific extraction processes aimed to prepare standardized extract for possible applications in herbal medicine or nutraceuticals.

## 4 Conclusion

This study on leaf extracts from *Z. budhensis* revealed significant bioactivities, in particular extracts presented significant effect as antimicrobial agent in vitro. The ethyl acetate and acetone extracts demonstrated the most significant antibacterial and antifungal activity among all the extracts tested and this can suggest a role by the triterpene acid fraction that is the most abundant in these extracts, combined to the flavan‐3‐ol derivatives as procyanidin and catechin that are also present in these two extracts. In addition, the extract was found to be safe thanks to the preliminary toxicological data obtained with brine shrimp test and the oral administration in vivo oral test on mice.

The phytochemical investigations allowed to establish the presence of multiple classes of phytoconstituents, namely flavan‐3‐ols, phenolic acids, flavonols, cyclopeptide alkaloids, triterpene acid, and triterpene saponins and the use of solvents with different polarity yielded in extracts with different compositions. The use of lipophilic solvents as hexane, dichloromethane and chloroform allowed the selective extraction of the triterpene acid. The more polar solvents starting from ethyl acetate, acetone and methanol resulted more efficient to extract both the lipophilic and hydrophilic constituents as glycosylated and non‐glycosylated polyphenols, cyclopeptide alkaloids, alkaloids triterpene aglycone, and saponins. This behavior was substantiated with the quantification of 23 different compounds observed in the plant material. The chemical analysis indicates that *Z. budhensis* is a valuable source of different phytochemicals. This study revealed the chemical constituents of *Z. budhensis* and also showed preliminary bioactivities in the area of antimicrobial effects that will need further evaluation to establish potential practical applications. Furthermore, the presence of complex pattern of phytoconstituents holds significant potential in pharmacology for developing lead compounds aimed at creating drugs to treat conditions such as diabetes and neurodegenerative diseases. The study indicates that hexane and dichloromethane extracts of *Z. budhensis* leaves exhibit nontoxic properties, suggesting their suitability for drug development.

## 5 Experimental Section

### 5.1 Collection of the Plant Materials

Leaves of *Z. budhensis* (synonym *Z. xiangchengensis* Y. L. Chen & P. K. Chou) were gathered in March 2021 from the Timal area of Kavrepalanchok District in Nepal at an elevation ranging from 1000 to 2000 m (27°32′44.52″ N, 85°38′00.96″ E). It was verified by Ms. Rita Chhetri, Research Officer, National Herbarium & Plant Laboratories (KATH) on August 8, 2024, and deposited with voucher specimen no. S‐202 (KATH).

### 5.2 Materials and Extraction Techniques

The leaves were rinsed with water and then dried in the shade. Once fully dried, they were ground into a powder using a grinding machine. 1.5 kg powdered leaves were transferred to a 5000 mL conical flask, and 3.5 L of hexane was added. The extraction was performed using an ultrasonic extraction process (40 kHz). Hexane was removed by filtration and concentrated using a rotary evaporator. The remaining residue of plant material was extracted with dichloromethane, and the same process was continued with chloroform, ethyl acetate, acetone, and methanol. Six extracts were prepared through sequential extraction with different solvents with increasing polarity, namely hexane, dichloromethane, chloroform, ethyl acetate, acetone, and methanol (called ZBLH, ZBLD, ZBLC, ZBLE, ZBLA, ZBLM, respectively). This sequential extraction guarantees the extraction of a large variety of phytochemicals with different polarities. Nonpolar solvents extract lipophilic (fat‐soluble) compounds whereas polar solvents extract hydrophilic (water‐soluble) compounds [45]. All the crude extracts obtained were concentrated till dryness, and the extract was stored at room temperature and carried out further processing.

The percentage yield was calculated by the given formula:

$$\%\mathrm{Yield}=\frac{\mathrm{Dry}\mathrm{weight}\mathrm{of}\mathrm{extract}}{\mathrm{Dry}\mathrm{weight}\mathrm{of}\mathrm{plant}}\;\times \;100\%$$

### 5.3 Phytochemical Screening

The leaf extracts of *Z. budhensis* were examined to identify the presence of different chemical constituents, such as alkaloids, flavonoids, phenolic compounds, carbohydrates, proteins, glycosides, steroids, tannins, terpenoids, and saponins. These analyses were carried out using the standard protocol [46, 47].

### 5.4 TPC

The Folin–Ciocalteu method was employed to assess the total concentration of phenolic compounds in all six *Z. budhensis* extracts. Gallic acid was fully dissolved in 1 mL of distilled water at a concentration of 1 mg. A 1 mL sample of 1 mg/mL gallic acid in methanol was mixed with 1 mL of Folin–Ciocalteu phenol reagent, which had been diluted 1:10 with water, and 0.8 mL of a 1 M Na_2_CO_3_ aqueous solution. The reaction mixture was left in the dark for about 15 min before measuring absorbance at 765 nm. Gallic acid served as the reference standard, and the TPC was reported as gallic acid equivalents per gram of dried extract [23, 48, 49].

Using the following method, the TPC in all samples was measured in milligrams of gallic acid equivalent:

$$\;\mathrm{TPC}=\frac{C\times V}{m}$$

where *C* is the concentration of gallic acid from curve (mg/mL), *V* is the volume of extract (mL), and *m* is the weight of plant extract (g).

### 5.5 TFC

The TFC in the *Z. budhensis* extracts was measured using the aluminum chloride colorimetric method. A 1 mL sample (0.1 mg/mL in methanol) was mixed with 1 mL of a 10% AlCl_3_ solution and allowed to stand for 1 h. The absorbance of the resulting mixture was then measured at 420 nm and compared to a control sample consisting of methanol only. Quercetin was used as the reference standard. The flavonoid concentration was calculated as milligrams of quercetin equivalent per gram of dry material [50]. The TFC of the sample was determined using this calculation:

$$\mathrm{TFC}=\frac{C\times V}{m}$$

where *C* is the concentration of quercetin from curve (mg/mL), *V* is the volume of extract (mL), and *m* is the weight of plant extract (g).

### 5.6 Antimicrobial Activity

#### 5.6.1 Preparation of Microbial Culture media

In 1 L of water, the liquid broth (LB) media was prepared by dissolving 13 g of LB powder (Sisco Research Laboratories Pvt. Ltd., India). The solution was autoclaving under a pressure of 15 psi at a temperature of 121°C for 25 min. The sterilized medium was chilled to a temperature range of 40°C–50°C and then transferred into 15 mL falcon tubes, with 5 mL of media in each tube. This prepared media was used to co‐culture bacterial seed cultures in separate tubes, which were then incubated for 24 h [51].

#### 5.6.2 Preparation of Mueller–Hinton Media Plates and Antimicrobial Assay

In 1 L of water the Mueller–Hinton agar (MHA) plates were made by dissolving 39 g of MHA powder (Sisco Research Laboratories Pvt. Ltd., India). The solution was autoclaved at 15 psi and 121°C for 25 min. After sterilization, the medium was cooled to 40°C–50°C and poured into Petri dishes, with each dish receiving 25 mL of the medium. The prepared plates were stored in a refrigerator until needed. Each plate was labeled with the sample names, and 150 µL of liquid bacterial seed was evenly spread across the surface using a sterile cotton swab. Wells were then made on the agar surface, and 100 µL of the samples dissolved in the medium at the concentration of 100 mg/mL and 10 µL of a 5 mg/mL kanamycin solution were added to the wells. The plates were incubated at 37°C for 24 h, and the results of the antimicrobial test were observed after this incubation period [26, 52].

### 5.7 Brine Shrimp Lethality Assay

To evaluate the cytotoxicity of bioactive compounds, tests were performed using brine shrimp (*Artemia salina*, also known as fairy shrimp or sea monkeys) to assess their lethality. The brine shrimp nauplii were exposed to solutions with varying concentrations of extracts from *Z. budhensis* for 24 h. The cytotoxic potential of these extracts was determined by counting the number of motile nauplii. The brine shrimp lethality assay indicates cytotoxicity if the LC_50_ value is below 1000 µg/mL [53, 54, 55, 56].

### 5.8 Antioxidant Activity

Antioxidant assays were performed by DPPH assay. Each extract (1.5 mg) was dissolved in methanol (1 mL) to create an initial stock solution (1.5 mg/mL). From this stock solution, various concentrations were prepared, including 1000, 500, 250, 125, 62.5, and 31.25 µg/mL, using a twofold dilution method. For the assay, 500 µL of each concentration was mixed with 1500 µL of a 0.1 mM DPPH methanol solution. The solutions were shaken vigorously for 2 min, and then the vials were covered with aluminum foil to prevent unexpected reaction.

The solutions were left at room temperature, protected from light, for 30 min. Following this period, the absorbance at 517 nm was measured using methanol as a reference. For the control, a mixture of 1.5 mL of DPPH solution and 0.5 mL of methanol was used, and its absorbance at 517 nm was recorded [57]. A calibration curve was also constructed.

The percentage of radical scavenging activity was calculated using the following formula:

$$\mathrm{Percentage}\;\mathrm{scavenging}=\frac{\left(A_{0}-A_{T}\right)}{A_{0}}\times 100\%$$

where *A*_0_ is the absorbance of the DPPH and *A*_T_ is the absorbance of the DPPH free radical solution containing the sample extract.

The IC_50_ value is the sample concentration essential to scavenge 50% of the DPPH free radicals. By utilizing the logarithmic dosage inhibition curve, we successfully determined the IC_50_ values by graphing the concentration of the extract against its matching scavenging effect [58, 59, 60].

### 5.9 In Vivo Acute Oral Toxicity Study

The OECD's Acute Toxic Class Method 425 was applied following the chemical testing guidelines for acute oral toxicity. The experiments were conducted on mice at the pharmacology lab of the Natural Product Research Laboratory (NPRL) in Thapathali, Nepal, experiments were approved by Department of Plant Resources, Natural Products Research, Government of Nepal, Ministry of Forest & Environment (2708081). Prior to the trial, the mice underwent a 12‐h fasting period. Their body weight was measured just before administering the extracts. The mice were randomly divided into two groups: the control group received physiological saline, while the experimental group was given the extract via an orogastric tube at a dosage of 2000 mg/kg of body weight.

The subjects were observed clinically four times a day, with close attention paid to their behavior, overall health, nasal mucosa, skin and fur condition, respiratory rate, somatomotor activity, and any potential symptoms such as tremors, convulsions, diarrhea, fatigue, and excessive salivation, reduced responsiveness to stimuli, changes in sleep patterns, light sensitivity, and coma. Abdominal palpation was also performed. After 48 h of clinical observation, during which no adverse effects were noted, the experimental group received a dosage of 2000 mg/kg of leaf extract. The “*t*‐test for independent groups” was used for statistical analysis in STATISTIC V. 7.0 for Windows, with a significance level set at *p* = 0.005. At the conclusion of the experiment, the mice were euthanized humanely [61, 62, 63].

### 5.10 LC‐DAD‐MS^n^, Liquid Chromatography QTOF Measurements

For LC‐DAD‐MS analysis, an Agilent 1260 chromatograph equipped with a 1260 series diode array was used (Agilent, Santa Clara, CA, USA). After the chromatography column, a “T” junction split the flow to DAD and Varian MS 500 ion trap mass spectrometer (Varian, Santa Clara, CA, USA). The spectrometer operated in negative ion mode, acquiring spectra in *m*/*z* 50–1500 range. Agilent SBC18 4.6 × 50 mm (1.8 micron) was used for the separation. The mobile phase was water 1%formic acid (A), acetonitrile (B), and methanol (C). A gradient program was used as follows: 0 ⟶ 0.5th min: A:B:C (95:5:0) ⟶ A:B:C (95:5:0), 0.5 ⟶ 5th min: A:B:C (95:5:0) ⟶ A:B:C (85:15:0), 5 ⟶ 15th min: A:B:C (85:15:0) ⟶ A:B:C (60:30:10), 15 ⟶ 20th min: A:B:C (60:30:10) ⟶ A:B:C(20:70:10), 20 ⟶ 25th min: A:B:C (20:70:10) ⟶ A:B:C (0:90:10), 25 ⟶ 30th min: A:B:C (0:90:10) ⟶ A:B:C(0:90:10), 30 ⟶ 31th min: A:B:C (0:90:10) ⟶ A:B:C (95:5:0), 31 ⟶ 37th min: A:B:C (95:5:0) ⟶ A:B:C (95:5:0). Flow rate was 0.750 mL/min. For qualitative purposes, spectra were acquired in negative ion mode and MS^n^ spectra were acquired using the tdds function of the instrument. Collected data were compared to the literature and used to identify the compound, finally identified compounds were compared with reference standards when available. DAD detector was set at 254, 280, 330, 350 nm. Accurate *m*/*z* values (ppm < 10) were obtained using a Waters Acquity H‐Class UPLC system coupled to a Waters Xevo G2 QTOF MS detector, operating in ESI (+) mode. The stationary phase was a Waters BEH C18 (2.1 × 50 mm) and eluents were water 0.1% formic acid (A) and acetonitrile 0.1% formic acid (B). Flow rate was 0.3 mL/min and gradient start with 90% A and in 15 min reach 90% B then in 5 min go to 100%B and stay isocratic up to 18 min than go back to initial conditions. MS parameters were as follows: sampling cone voltage, 40 V; source offset, 80 V; capillary voltage, 3500 V; nebulizer gas (N2) flow rate, 800 L/h; desolvation temperature, 450°C. The mass accuracy and reproducibility were maintained by infusing lockmass solution thorough Lockspray at a flow rate of 20 µL/min. Centroided data were collected in the mass range 50–1200 Da, and the *m*/*z* values were automatically corrected during acquisition using lockmass. Accurate *m*/*z* values of fragments were acquired performing a parallel MS/MS experiment, keeping the collision energy constant at 30 V.

For quantification of the selected compounds the following reference compounds were used namely catechin, procyanidin B2, rutin, quercetin, kaempferol‐3‐O‐glucoside, kaempferol, and maslinic acid. Standard solutions were prepared for the calibration curves in the concentration range from 50 to 5 µg/mL. *Z. budhensis* dried extracts were weighed (50 mg) with accuracy of ±0.1 mg and methanol was added in small volume. Samples were sonicated for 5 min and then further methanol was added and sonication repeated. Finally, volume was adjusted in volumetric flask of 25 mL. The solution was centrifuged, and supernatants were used for the analysis.

### 5.11 Statistical Analysis

Related to the in vivo test, the “*t*‐test for independent groups” was used for statistical analysis in STATISTIC V. 7.0 for Windows, with a significance level set at *p* = 0.005. At the conclusion of the experiment, the mice were euthanized humanely [61, 62, 63].

Related to the antioxidant activity the experiments were performed in triplicate, and differences between the extracts were compared using one‐way ANOVA (by Tukey's assay) and GraphPad Prism (version 9.2) was used for the analysis. The *p* value of less than 0.05 was considered as statistically significant. Considering the quantitative data the values are expressed as mean ± standard deviation.

## Author Contributions

**Samjhana Bharati**: conceptualization, lab work, manuscript preparation, review and editing. **Binita Maharjan**: manuscript preparation, review and editing. **Timila Shrestha**: lab work. **Shyam Sharan Shrestha**: review and editing. **Stefania Sut**: conceptualization, manuscript preparation, review and editing, supervision. **Hari Prasad Devkota**: conceptualization, review and editing, supervision. **Ram Lal Swagat Shrestha**: conceptualization, review and editing, manuscript preparation, supervision. **Stefano Dall'Acqua**: conceptualization, manuscript preparation, supervision, review and editing.

## Conflicts of Interest

The authors declare no conflict of interest.

## Data Availability

The data that support the findings of this study are available from the corresponding author upon reasonable request.
